# Resistive Switching in All-Printed, Flexible and Hybrid MoS_2_-PVA Nanocomposite based Memristive Device Fabricated by Reverse Offset

**DOI:** 10.1038/srep36195

**Published:** 2016-11-04

**Authors:** Muhammad Muqeet Rehman, Ghayas Uddin Siddiqui, Jahan Zeb Gul, Soo-Wan Kim, Jong Hwan Lim, Kyung Hyun Choi

**Affiliations:** 1 Department of Mechatronics Engineering, Jeju National University, Jeju, Republic of Korea

## Abstract

Owing to the increasing interest in the nonvolatile memory devices, resistive switching based on hybrid nanocomposite of a 2D material, molybdenum disulphide (MoS_2_) and polyvinyl alcohol (PVA) is explored in this work. As a proof of concept, we have demonstrated the fabrication of a memory device with the configuration of PET/Ag/MoS_2_-PVA/Ag via an all printed, hybrid, and state of the art fabrication approach. Bottom Ag electrodes, active layer of hybrid MoS_2_-PVA nanocomposite and top Ag electrode are deposited by reverse offset, electrohydrodynamic (EHD) atomization and electrohydrodynamic (EHD) patterning respectively. The fabricated device displayed characteristic bistable, nonvolatile and rewritable resistive switching behavior at a low operating voltage. A decent off/on ratio, high retention time, and large endurance of 1.28 × 10^2^, 10^5^ sec and 1000 voltage sweeps were recorded respectively. Double logarithmic curve satisfy the trap controlled space charge limited current (TCSCLC) model in high resistance state (HRS) and ohmic model in low resistance state (LRS). Bendability test at various bending diameters (50-2 mm) for 1500 cycles was carried out to show the mechanical robustness of fabricated device.

Graphene was the first two dimensional (2D) material to be found with intriguing electrical properties and unique chemical structure that led to the discovery of various materials of its kind like the transition metal dichalcogenides (TMDs)^1^. These 2D materials have shown attractive electronic properties for electronic device applications like OLED, optoelectronics, TFT and sensors^2^,^3^,^4^,^5^. In recent years quasi 2D materials like WSe_2_, WS_2_, MoS_2_ and MoSe_2_ are under intensive research as they also have a unique potential to be used in nanotechnology and nanoscience for various applications. Chemically these materials are poised of two dimensional set of sandwiched sheets. Interlayer atoms of stacked layers are covalently bonded to each other while weak Van der Waals forces are responsible for holding these two layers together^6^.

Among these 2D materials, MoS_2_ is a very attractive candidate with an immense potential to be used in electronic device applications because of its suitable quantum confinement, high mobility owing to direct band gap of 1.85 eV in monolayer, and indirect bandgap of 1.29 eV in multilayer^7^. MoS_2_ has 1T (metallic phase), 2H (hexagonal symmetry), 3R (rhombohedral) and other phases due to single, double, triple and multilayers stacked over each other respectively. Sulfur-molybdenum-sulfur having hexagonal structure are sandwiched over one another. Owing to the quick progress of 2D materials to be used in future device applications, various techniques to synthesize monolayer and a few layer MoS_2_ flakes have been explored. These methods include micromechanical exfoliation, hydrothermal process, solution-based chemical exfoliation, chemical vaporization, ion intercalation exfoliation, and sulfurization of molybdenum (Mo)^8^,^9^,^10^,^11^,^12^. Exfoliated MoS_2_ semiconductor has shown remarkable electrical properties and mechanical strength in various micro electronic device applications such as lasers^13^, supercapacitors^14^, thin film transistors (TFTs)^15^, phototransistors^16^, bio sensors^17^, solar cells^18^, and batteries^19^.

Although various contributions have already been made to use MoS_2_ in various electronic device applications as mentioned above, but the resistive switching characteristics of this 2D material in the form of a memristor in particular has yet to be completely explored. A memristor is the fourth basic element which was realized in 2008 by HP laboratories^20^. It has a very low operating voltage, extremely simple sandwich structure and remarkably small size unlike traditional transistors. A lot of research is being conducted to explore the unique nanocomposite materials with memristive effect owing to its potential of replacing transistor from memory devices^21^,^22^,^23^,^24^,^25^. Nanospheres, monolayer, and multilayer flakes of MoS_2_ have recently been reported to show remarkable memristive properties with a very high off/on ratio of up to 10^6^ and reproducibility of 10^4^ voltage sweeps^6^,^7^,^26^,^27^,^28^. Juqing Liu *et al*.^28^ and Sachin M. Shinde *et al*.^7^ have recently reported appreciable results of resistive switching by using a combination of exfoliated MoS_2_ flakes with a polymer.

In this work we have extensively explored the resistive switching characteristics of a novel hybrid nanocomposite synthesized by coating exfoliated few layered semi-conductive MoS_2_ flakes with a dielectric PVA polymer on a flexible polyethylene terephthalate (PET) substrate. MoS_2_ was selected owing to its graphene like 2D structure with promising semiconducting properties. MoS_2_ is highly suitable material to trap charge carriers due to its appropriate quantum confinement and available energy states. PVA is used as an isolating dielectric material and a supportive layer to transfer the MoS_2_ flakes onto the bottom electrodes. Liquid exfoliation method was conducted to exfoliate pristine MoS_2_ flakes into a few layered and to dissolve PVA in N-Methyl-2-pyrrolidone (NMP) solvent. Structure of PVA shows that it is a branched polymer, significantly suitable for coating a 2D material to form a highly uniform thin film. Moreover PVA is a non-toxic, biocompatible, environment friendly, cheap and non-hazardous organic polymer. This work was undertaken to comprehensively explore the resistive switching effect in a novel nanocomposite of MoS_2_-PVA as being the potential candidate for the next generation memory device. The fabricated device exhibited a rewritable, non-volatile and bipolar resistive switching.

## Results and Discussion

### Morphological Characterization

**Figure 1:**
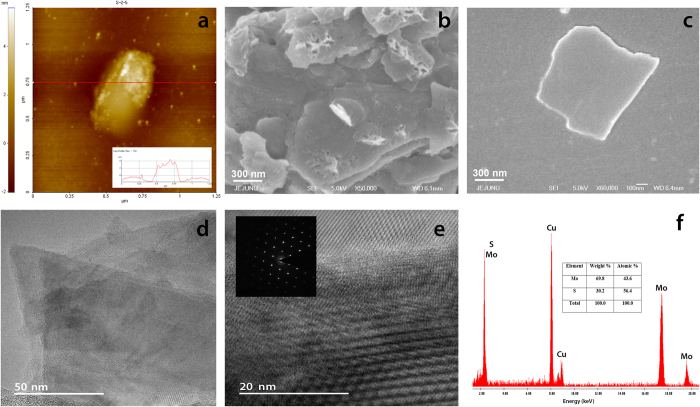
AFM, FESEM and TEM images of as synthesized MoS_2_ flakes. (**a**) AFM image of single exfoliated MoS_2_ flake illustrating its few layered nature with average thickness of 4 nm. (**b**) FESEM image illustrating the high densityof as synthesized MoS_2_ flakes. (**c**) A single exfoliated flake of MoS_2_. (**d**) Typical bright field TEM image of MoS_2_ flakes showing layer edges. (**e**) Phase contrast High Resolution (HR) TEM image of thin MoS_2_ flakes clearly displaying layer edges. Inset in (**e**) shows the selective area electron diffraction (SAED) pattern. (**f**) Energy dispersive X-ray spectroscopy (EDS) of exfoliated MoS_2_ flakes. Scale bar of above images have been redrawn for better visibility.

In order to analyze the morphological studies of as synthesized few layered MoS_2_ flakes with varying thickness depending upon the number of layers, transmission electron microscope (TEM), field emission scanning electron microscope (FESEM) and atomic force microscope (AFM) images were recorded in Fig. 1. These images show that regions with same thickness (i.e. same number of layers) have identical contrast. The image taken by AFM illustrates a few layered nature of exfoliated MoS_2_ flakes as the average thickness is 3 nm which is equivalent to 3–4 layers as shown in Fig. 1(a). The images taken from FESEM at a high resolution show extremely high density of sprayed MoS_2_ flakes with no gaps in between them as shown in Fig. 1(b). These images show a chaotic array of MoS_2_ flakes stacked mostly in plane. We attained very thin yet unbroken MoS_2_ flakes film with conventional lateral dimensions. SEM image of a single exfoliated flake of MoS_2_ is shown in Fig. 1(c). The prepared MoS_2_ flakes were further critically characterized by taking images from Transmission Electron Microscope (TEM). TEM images also indicated the existence of a few layered exfoliated MoS_2_ flakes with varying thickness. The regions with darker shade have more thickness owing to more number of layers as compared to regions with light color. Edges of individual layers stacked over one another can easily be observed near the periphery of MoS_2_ flakes in High Resolution (HR) TEM image of Fig. 1(d). HRTEM image is clearly exhibiting the layered structure of as synthesized MoS_2_ flakes. The distance between two consecutive layers as seen from the image of Fig. 1(e) is evidently less than 1 nm. Inset of Fig. 1(e) displays the selective area electron diffraction (SAED) pattern that is consistent with a plain lattice showing that the prepared MoS_2_ flakes are highly crystalline. Elemental analysis of exfoliated MoS_2_ sample is conducted by using the energy dispersive X-ray spectroscopy (EDS) showing the existence of Mo and S as depicted by Fig. 1(f). The surface morphology of as deposited MoS_2_-PVA nanocomposite thin film was measured by 2D-Nanomaping as shown in Supplementary Fig. S1. Values of average surface roughness (Ra) and Root mean square roughness (Rq) were 41.19 nm and 71.23 nm respectively.

### Optical Characterization

**Figure 2:**
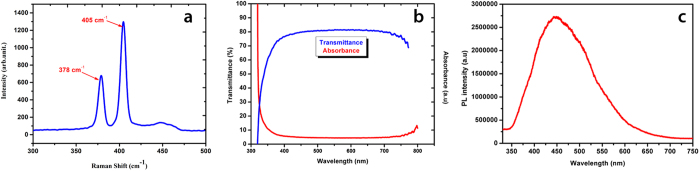
(**a**) Typical Raman spectra of a few layered MoS_2_ semiconductor (**b**) UV-vis optical transmittance spectra of the as synthesized few layered MoS_2_ flakes (**c)** Photoluminescence (PL) spectra of MoS_2_ nanosheets.

Raman spectroscopy analysis was carried out to conform the conversion of few layer formation from pristine MoS_2_ flakes. It is a very useful technique for quantitatively measuring the number of layers present in exfoliated MoS_2_ flakes. Raman spectroscopy utilize inflexible scattering of homochromatic light from a laser. The energy of the photons shifts upward or downward depending upon the laser interacting with vibrations in the system. Low power laser is desirable in order to protect sample from disintegration. Raman spectroscopy was necessary for ensuring the existence of MoS_2_ flakes in the prepared solution. Figure 2(a) illustrates the Raman spectra of exfoliated MoS_2_ and signifies the presence of its few layered flakes. Raman spectra was plotted in the range of 300 cm^−1^ to 500 cm^−1^. The characteristic peaks of a few layered MoS_2_ representing E_2g_^1^ and A_1g_ were found at 378 cm^−1^ and 405 cm^−1^ respectively which is in agreement with already reported literature^29^. Peak of E_2g_^1^ is due to in plane vibrations between two sulfur (S) and a single molybdenum (Mo) atoms in the basal plane whereas peak of A_1g_ is due to out of plane vibrations of only S atoms along c axis relative to each other. These two peaks undisputedly suggest that the structure of prepared MoS_2_ flakes is 2H^30^.

In order to determine the optical properties of as synthesized MoS_2_ flakes, wavelength dependent transmittance characterization was carried out by UV/Vis spectroscopy in the range of 300–800 nm as shown in Fig. 2(b). An optical transmittance of ≈80% was observed in the as synthesized MoS_2_ flakes. The absorbance curve is also shown along with the transmittance curve for better understanding. This further conforms the presence of a few layered MoS_2_ structure because transmittance value of a monolayer MoS_2_ flake is much higher (nearly 95%)^31^. Total number of layers and the transmittance of MoS_2_ flakes is inversely proportional to each other. The transmittance remained nearly constant from 400 nm to 770 nm and began to decrease after 770 nm. Visible light is absorbed and reflected several times in a few layered structure.

Figure 2(c) illustrates the emission spectrum of as synthesized MoS_2_ flakes recorded by photoluminescence spectroscopy (PL). A broad peak was detected at a wavelength of 440 nm. UV/Vis spectroscopy further endorse the presence of a few layered MoS_2_ flakes because for monolayer MoS_2_ nanosheets this broad peek appears at nearly 652 nm. The difference in the peak value is due to direct and indirect bandgap in monolayer and a few layer MoS_2_ flakes respectively. This might be due to quantum confinement effect across the 2D planes^29^. When 2D materials are exfoliated by mechanical approach like scotch tape method, it produces maximum flakes with large lateral size and its photoluminescence peak appears at 652 nm but when grinding and ultrasonication method is adopted different sizes of nanosheets are produced including small quantity of quantum dots and photoluminescence whose peak appears at 440 nm. This quantum confinement effect is due to the formation of MoS_2_ quantum dots along with a few layered flakes as illustrated in Fig. 1(a). All characterizations presented in Fig. 2 were performed at room temperature and ambient conditions.

### Electrical Characterization

**Figure 3:**
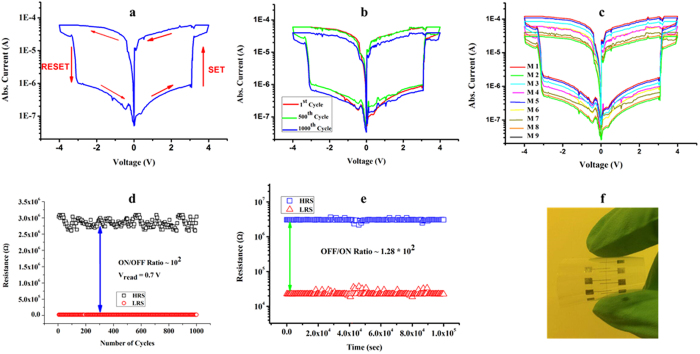
Electrical characterization of memory device. (**a**) Typical log I-V characteristic curve of a memristor showing bipolar resistive switching. (**b**) Endurance measurement showing excellent repeatability of 1^st^, 500^th^, and 1000^th^ cycle. (**c**) Typical I-V curves showing the bipolar resistive switching effect in all the 9 memory cells of a 9 × 1 array labeled as M1-M9 respectively. (**d**) Endurance of HRS and LRS at a reading voltage of 0.7 volt showing remarkable stability. (**e**) Retention of HRS and LRS measured over a longer period of time without any noticeable deviation at 0.7 volts. (**f**) Optical Image of as fabricated memory device on a flexible PET substrate in bend condition.

I-V characterization of MoS_2_-PVA based memory devices was done by using Aglient B1500A Semiconductor Device Analyzer. Biased voltage was applied to the top Ag electrode while the bottom Ag electrode was grounded. Current compliance (C.C) value was set as 60 uA in the applied voltage sweep range of −4 to +4 volts. The typical characteristic semi-logarithmic I-V curve of resistive switching was observed in our memory device based on MoS_2_-PVA nanocomposite as displayed in Fig. 3(a). Current slightly kept increasing with increasing value of voltage while maintaining its high resistance state (HRS) until the applied voltage reached to a threshold value (V_th_) of ≈3 V. The low threshold voltage can be attributed to the high conductivity of Ag electrodes assisted by charged MoS_2_ flakes. Current abruptly increased from 9.6 × 10^−7^ to 8.5 × 10^−5^ A, resulting in a clear resistive switching from HRS to low resistance state (LRS). It can be deduced from this information that data can be written on this memory device at a voltage of ≈3 V. The tunable electrical resistance exist in the range of 22 kΩ to 3 MΩ.

**Figure 4:**
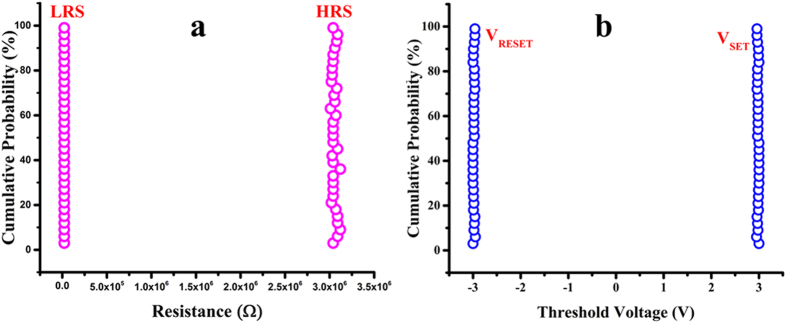
Cumulative Probability plot displaying highly stable and repeatable experimental values of both resistive states and threshold voltages for storing and removing data. (**a**) LRS and HRS (**b**) Set and RESET voltage for writing and erasing data.

A decent off/on ratio at a read voltage (V_read_) of 0.7 V was recorded to be equal to 1.28 × 10^2^ that is enough to distinguish HRS from LRS. Figure 3(b) illustrates excellent endurance for both HRS and LRS when tested against 1000 voltage cycles as both resistive states showed extremely high stability. Our fabricated memory device comprise an array of nine memory cells. Characteristic logarithmic curve of all the nine memory cells was plotted against each other that showed significant repeatability as shown in Fig. 3(c). The highly symmetric nature of characteristic I-V curves was due to the selection of same Ag metal as the top and bottom electrode with a single uniform nanocomposite layer. Figure 3(d) illustrates the endurance of both resistive states i.e. HRS and LRS over 1000 voltage cycles displaying excellent repeatability. Once the bits were written and external power source was removed, the device retained the stored data for over 10^5^ sec without any noticeable decline in HRS or LRS, demonstrating its outstanding non-volatile memory behavior as shown in Fig. 3(e). Optical image of real device in bend state is shown in Fig. 3(f). Both resistive states i.e. HRS and LRS of MoS_2_-PVA based memory device are highly uniform as displayed in Fig. 4(a). Data can be stored and erased from the memory device by applying very small value of set and reset voltage. Cumulative plot of threshold voltage illustrates repeatable values for both V_SET_ and V_RESET_ as shown in Fig. 4(b).

### Conduction Mechanism

**Figure 5:**
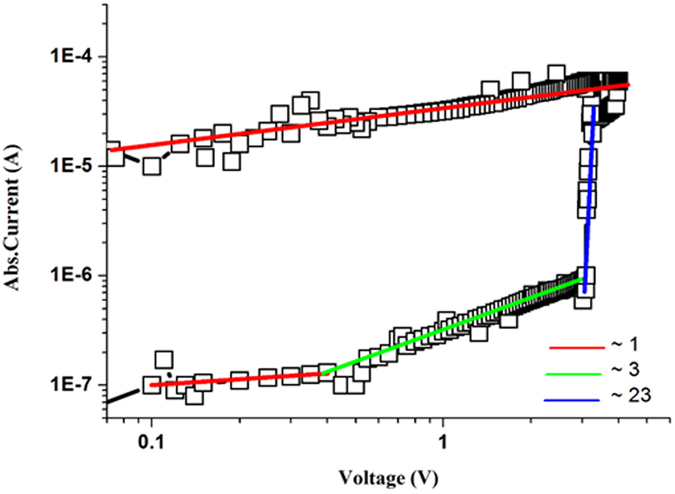
Double Logarithmic I-V curve showing the slopes for HRS and LRS with different values of slope. This plot can be divided into three distinguish regions as separated by the three different colors. In the red line region, ohmic conduction takes place as slope ≈1. The green line is the Child’s law region as the slope value ≈3. The blue line shows the region of sharp current increase as slope ≈23 where the device switches from HRS to LRS.

In order to understand the conduction mechanism taking place in our device, model fitting studies for both HRS and LRS was carried out by taking logarithm of both current (y-axis) and voltage (x-axis) as shown in Fig. 5. This plot suggests that LRS is governed by ohmic conduction while HRS is governed by a typical trap controlled space charge limited current (TCSCLC) model as its plot can be confined by three restricted curves based on the slope values. These results are comparable to already reported conduction mechanism for MoS_2_/PMMA functional thin film by Shinde *et al*.^7^. In region I (0–0.4 V), majority of charge carriers occur due to thermionic emission owing to lower value of applied bias voltage resulting in ohmic behavior (slope ≈1). These charge carriers mainly depend on the magnitude of externally applied electric field and electronic properties of functional materials^32^,^33^. As the applied electric field increases, electrons from the metallic electrodes gain enough energy to jump into the active nanocomposite layer of MoS_2_-PVA. Region II (0.4–3 V) signifies that as magnitude of applied voltage increase, traps present within the active layer of MoS_2_-PVA functional layer begin to partially fill with charge carriers hence slightly increasing the conductivity (slope ≈3) as suggested by Son *et al*.^32^,^33^. In region III (3–4 V), the magnitude of applied electric field is further increased and all the available unoccupied energy levels or traps are fully filled by the charge carriers resulting in an exponential increase in current as I ∝ V^m^ (slope ≈23)^32^,^33^. This sharp increase in the current value can be associated with the exponential spread of trap states within a large bandgap between conduction and valence band of PVA polymer. On the other hand, initially the conduction band of MoS_2_ flakes is partially filled with electrons. All the electrons present in the conduction band of MoS_2_ have complete freedom of movement. As applied electric field is increased, electrons from the valence band of MoS_2_ gain enough energy to jump into the conduction band and fill the unoccupied energy levels. The number of electrons in the conduction band of MoS_2_ continue to increase with increasing applied bias voltage, hence resulting in higher conductivity of the memory device. This turns the device from HRS to LRS state. The conduction of charges in the LRS is governed by ohmic conduction (I ∝ V) owing to the value of linear fit line (slope ≈1) indicating the presence of good conductive channels.

Choice of the metal contact is very critical for memory device as it determines the height of the energy barrier for the injected electrons from electrode into functional thin film. It is a fact that lower the value of work function, lower will be the energy barrier heights, resulting in a large charge carrier flow from metal contacts into active thin film. Ag metal was chosen as the top and bottom electrode owing to its lower work function (4.26 eV) relative to the electron affinity of MoS_2_ as compared to other metal electrodes like Pt (5.70 eV), Au (5.40 eV) and tungsten (5.1 eV)^34^. Several energy states are present within the band gaps of MoS_2_ semiconductor and PVA polymer. Primarily, the charge carriers are present in energy states with lower mobility keeping the device in HRS condition. In our memory device, energy states with lower energy levels are offered by exfoliated MoS_2_ flakes. Electrons are trapped in the potential wells of MoS_2_ due to its quantum confinement and lower energy levels. Structure of our device can be attributed to an assembly of potential wells with different widths, depths and asymmetry’s owing to the nanocomposite of n-type semiconducting MoS_2_ flakes and dielectric PVA polymer as the functional materials. This difference is due to different magnitude of work function, HOMO and LUMO energy levels. Furthermore the states are localized in potential wells due to which movement of charge carriers takes place by hopping mechanism.

Before the application of voltage sweep, charge carriers trapped in the potential wells are in Fermi level. Initially charges are present in lowest available energy levels where they have low mobility. On the application of electric field, charge carriers gain enough energy and bending of energy barriers takes place. These excited electrons begin to flow from lower energy states into traps with higher energy. This partially fulfills the traps and conductivity of device begins to increase. As the magnitude of external applied electric field is further increased, its value reaches the threshold voltage (V_th_) at which all the traps with higher mobility are completely filled with charge carriers and the memory device switches to LRS. The huge bandgap offered by PVA polymer due to its dielectric property makes it extremely difficult for the trapped charge carriers to return to their original energy states without the aid of an external power source. This insulating property of PVA restricts the flow of trapped charge carriers back to their original energy states with lower mobility resulting in remarkably high retention time. In order to turn the device back to its original resistance state of HRS from LRS an external voltage supply of opposite polarity is applied. As a result, trapped charge carriers gain enough energy to overcome the huge energy barrier offered by PVA. This turns the device back to its original resistive state of HRS. In this fabricated device, the composition of MoS_2_-PVA nanocomposite along with the choice of top and bottom electrodes is yet to be optimized which will surely further increase the efficiency of this device.

### Mechanical Robustness

**Figure 6:**
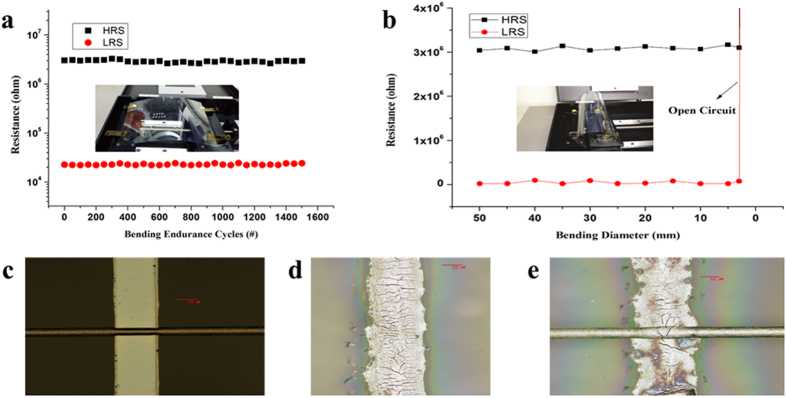
Bendability measurements by using the in-house prepared bending machine showing the remarkable flexible properties of as prepared resistive switching device. (**a**) Endurance curve showing HRS and LRS values for 1500 bending cycles at a fixed diameter value of 10 mm. (**b**) Endurance plot clearly showing very less deviation in HRS and LRS with changing values of bending diameters in the range of 30 mm to 2 mm. The device behaved like an open circuit at a bending diameter of 2 mm. Optical images of as top and bottom electrodes at different values of bending diameters. (**c**) Before bending. (**d**) Appearance of micro-cracks in bottom electrode. (**e**) Development of a major crack in the bottom electrode and completely broken top electrode at a bending diameter of 2 mm resulting in the open circuit of memory device.

As the device was fabricated on a flexible PET substrate therefore its mechanical robustness was examined against several bending cycles by squeezing the PET substrate in forward and backward direction. An in-house designed bending machine was used for repeatable bendability test at desired curvatures. The device showed excellent mechanical strength against 1500 bending cycles without any considerable change in both resistive states at a fixed bending diameter of 10 mm as no considerable difference in the bi-stable resistive states was observed in Fig. 6(a). Inset of Fig. 6(a) illustrates the optical image of as fabricated device without bending. The resistance of both HRS and LRS was plotted against decreasing value of bending diameter ranging from 50 mm to 2 mm. Figure 6(b) shows the endurance of both resistive states i.e. HRS and LRS against several bending diameters exhibiting negligible deviation owing to the remarkable mechanical strength of these devices. Inset of Fig. 6(b) illustrates the optical image of as fabricated memory device in bend state. However bending beyond 2 mm caused the disintegration of electrodes that ruptured the path of electron flow resulting in open circuit. The formation of micro and major cracks with increasing bending diameter are shown in Fig. 6(c–e). These bendability test shows that MoS_2_-PVA based memory device is remarkably suitable to be used in flexible electronics.

The nonvolatile memory device based on hybrid MoS_2_-PVA nanocomposite exhibited superior electrical and mechanical characteristics such as better off/on ratio, high retention time, lower current compliance, larger endurance, low operating voltage and remarkable mechanical robustness when compared with already reported resistive switching devices based on PVA nanocomposites with other materials. Electrical characteristics of already reported PVA based memory devices have been compared with our device in Table 1. Moreover, the electrical characteristics of already reported MoS_2_ based memory devices have also been separately compared with MoS_2_-PVA based memory device reported in this work as shown in Table 2. Off/on ratio of MoS_2_-PVA nanocomposite is comparable to other already reported MoS_2_ based memory devices, however retention of this device is significantly higher owing to the high dielectric value of PVA which restricts the charge carriers to move back to their original energy states after the removal of external power supply. From Tables 1 and 2, it is noticeable that the results for mechanical robustness of either PVA or MoS_2_ based nanocomposite memory devices by bending at various diameters and for several bending cycles have never been reported before this work.

## Conclusion

Our work has demonstrated resistive switching effect of a hybrid organic-inorganic nanocomposite of a few layered MoS_2_ flakes passivized with a PVA polymer matrix. Pristine MoS_2_ flakes were exfoliated by using a facile and eco-friendly method of liquid exfoliation. A relatively new, yet extremely competitive printing system known as reverse offset was employed and described in detail to deposit high resolution bottom electrodes. The all printed memory device, with the configuration Ag/MoS_2_-PVA/Ag exhibited excellent non-volatile and rewritable bipolar resistive switching behavior. Data can be written and erased by applying a positive and negative bias of ≈3 V and ≈−3 V respectively. A high off/on ratio of 1.28 × 10^2^ was recorded which is enough to distinguish between HRS and LRS. Significantly high retention time of ~10^5^ sec and endurance of >1000 voltage sweeps was recorded at a reading voltage of 0.7 V. Results were highly repeatable for all the nine memory cells in the fabricated 9 × 1 array. Obtained results of MoS_2_-PVA based memory device were superior to many of its predecessor composites of either MoS_2_ or PVA. Moreover, mechanical robustness of MoS_2_ and PVA based memory device has also been reported for the first time in this work. The hybrid memory device displayed excellent durability and mechanical strength when tested against several bending cycles. The presented results broaden and deepen the understanding of memory phenomena in hybrid nanocomposites of 2D materials and polymers. Resistive switching properties of this functional hybrid nanocomposite can be further improved by optimizing its formulation and selection of electrode materials for future work. Our device has ample potential to be used as a nonvolatile memory device in various flexible electronic devices.

## Methods

### Materials

**Figure 7:**
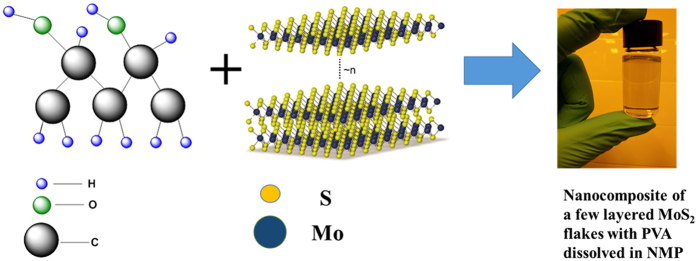
Chemical structures of dielectric PVA polymer and semi-conductive pristine MoS_2_ displaying their interatomic bonding along with the as synthesized exfoliated few layered MoS_2_ flakes ink with PVA dissolved in NMP solvent.

Pristine MoS_2_ (99.0%) ultrafine powder was purchased from Graphene Supermarket. PVA (M_W_ 9000–10000, 80% hydrolyzed) and NMP (anhydrous, 99.5%) were purchased from Sigma Aldrich. Ag nanoparticles ink (300 cps, 60 wt %) for depositing top and bottom electrodes were purchased from Paru Co.Ltd. Liquid exfoliation method was used for the synthesis of MoS_2_ flakes compared to mechanical method of scotch tape micro cleavage to achieve high throughput. Lateral flake size and thickness of raw flakes are affected by the selected exfoliation process. NMP was selected as the solvent for dispersing MoS_2_ in PVA. NMP is a suitable choice for exfoliating MoS_2_ powder due to its surface energy that matches well with the nanosheets of MoS_2_. The surface tension helps in overcoming the van der Walls forces resulting in ease of exfoliation. Grinding of pristine MoS_2_ powder in NMP was carried out for 1 hour in a mortar followed by heating at 100 °C for 1 hour to evaporate the solvent. Furthermore ground MoS_2_ was dispersed in NMP for probe sonication for 1 hour as grinding and sonication both are effective in exfoliation of MoS_2_ flakes^42^. Finally centrifugation at 6000 rpm was carried out for 30 min to remove the larger nanoplatelets and partially exfoliated crystallites. The supernatant was collected and PVA (10 wt. %) was dissolved in it. The blend solution of MoS_2_ and PVA was stirred on a magnetic stirrer at 1000 rpm at ambient conditions for overnight to observe if there are some undissolved suspensions left. The resulting homogeneous and uniformly dispersed nanocomposite of MoS_2_ with PVA in NMP was used for device fabrication. The dispersion remained stable over a period of several months and no suspension or segregation was observed. The molecular structures of PVA and MoS_2_ along with the resulting hybrid nanocomposite ink bottle are shown in Fig. 7.

### Patterning of bottom electrodes printed by reverse offset

**Figure 8:**
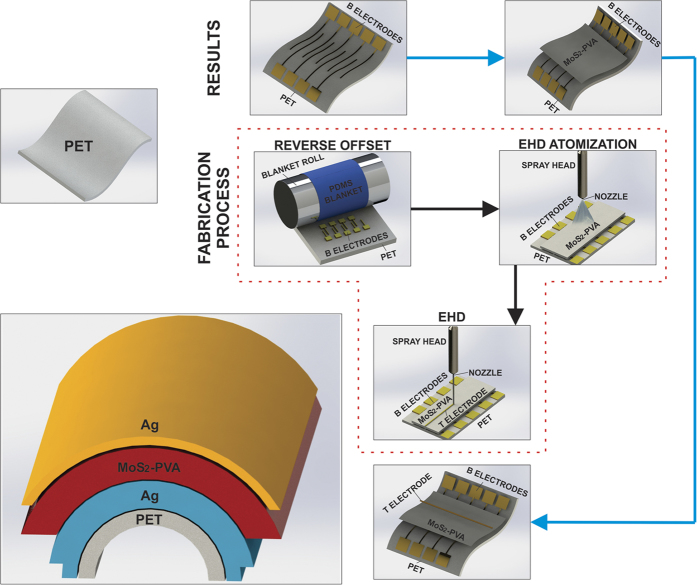
Schematic diagram illustrating the whole manufacturing process of as fabricated memory device on a flexible PET substrate. The images enclosed in the red dotted block exhibit each step of the fabrication process while the images outside the block correspond to the resulting device after each step. The bottom left corner exhibit layer by layer sandwiched structure of a single memory cell in bend state with PET/Ag/MoS_2_-PVA/Ag configuration.

Moon *et al*. have recently reported a novel direct contact method known as reverse offset printing for patterning high quality metal electrodes with the ability to print extremely tiny and complicated structures with uniform thickness^43^. In order to achieve fine patterns with high resolution, more accurate micro-fabrication technology is required. To realize such highly conductive electrodes, reverse offset printing is used in this work. Electrodes printed by reverse offset roll-to-plate printing technique have the promising characteristics of high resolution, low resistivity, high quality surface, simplicity, and ease of fabrication to realize an accurate and precise pattern^44^. It is a direct printing method that can print electrodes with pattern size <1 um unlike its other competitive direct printing techniques such as screen printing, flexographic printing, gravure printing and inkjet printing. The last step of reverse offset patterning known as SET process is illustrated in Fig. 8. A detailed explanation of reverse offset pattering process is provided in the Supplementary information with an illustration of every step in Supplementary Fig. S2.

### Deposition of MoS_2_-PVA nanocomposite by electrohydrodynamic atomization (EHDA)

Thin film of MoS_2_-PVA nanocomposite was successfully deposited by using an in-house built EHDA system. EHDA was preferred due to its high control, simplicity and ability to produce very thin films with high uniformity in quick time^45^,^46^. Complete schematic diagram of EHDA system with labelling of each part is shown in Supplementary Fig. S5. The MoS_2_-PVA nanocomposite solution was allowed to flow through the meniscus of the nozzle by applying the electric field as shown in the schematic diagram of Fig. 8. The flow rate was varied from 20 ul/hr–1000 ul/hr in order to achieve the stable cone jet for deposition of uniform thin film in desired area of target substrate. Hybrid MoS_2_-PVA nanocomposite ink being sprayed began to split into small droplets with increasing voltage. Spraying modes varied from dripping to multi-stable jet after passing through micro-dripping, unstable jet and stable jet. The spraying mode was controlled by the parameters of applied voltage, distance of nozzle from the substrate, nozzle diameter and set value of flow rate. The operating envelope of depositing MoS_2_-PVA nanocomposite through EHDA is shown in Supplementary Fig. S5. Film thickness was controlled by optimizing the number of spraying passes, spraying time, speed of stage and standoff distance between the nozzle and desired substrate. The values of all optimized parameters are listed in Supplementary Table S1 while the voltage range for achieving different modes of deposition are listed in Supplementary Table S2. Deposited thin film of MoS_2_-PVA nanocomposite was sintered at 120 °C for 2 hours in a furnace to evaporate its solvent completely. Optical images of EHD Atomization printing system and MoS_2_-PVA active layer on fabricated device is shown in Supplementary Fig. S4.

### Deposition of top electrode by electrohydrodynamic (EHD) patterning method

Finally the top Ag electrode was deposited by again using Ag nanoparticles ink. This task was performed via employing a non-contact technique by using the in-house developed electrohydrodynamic (EHD) patterning system. EHD patterning was preferred over other contact based printing techniques like screen printing, reverse offset and gravure offset because they could have damaged the MoS_2_-PVA based thin film resulting in a short circuit between top and bottom electrodes. However, on the other hand EHD patterning is a non-contact method with no such disadvantage. A single electrode of Ag nanoparticles ink with 30 μm width and 9 mm length was successfully deposited as the top electrode without damaging active thin film or bottom Ag electrodes as illustrated in Fig. 8. The optimized parameters during the deposition of top Ag electrode are listed in Supplementary Table S3. Top Ag electrode was sintered at 110 °C for 1 hour. The deposited electrode was highly conductive as its measured resistivity was only 0.2 ohm cm^−1^. Total area of as fabricated Ag/MoS_2_-PVA/Ag single memory cell was 30 μm × 100 μm. Optical image of EHD patterning system and top electrode on fabricated device is shown in Supplementary Fig. S4.

### Characterization

Raman spectra was recorded on (LabRAM HR Raman) spectrometer with a solid state laser at room temperature with the excitation laser wavelength of 514 nm. The UV/Vis spectrophotometer (Shimadzu UV 3150PC) was used to measure transmittance spectra ranging from 300 nm to 800 nm. Photoluminescence (PL) spectra were recorded by a (LS-55 Perkin Elmer Fluorescence Spectrometer) ranging from 350 nm to 700 nm. Field emission scanning electron microscope (FESEM) images were taken by using (Zeiss Supra 55VP) operating at 20 kV. The semiconductor device analyzer (Agilent B1500A) was used to measure the electrical characteristics. Optical images of printed electrodes were taken from (Olympus BX51M) computerized HR digital colored microscope. The 2D and 3D profiles of the film was observed using (NanoView NV2200) high accuracy 3D nano optical non-contact surface profiler.

## Additional Information

**How to cite this article**: Rehman, M. M. *et al*. Resistive Switching in All-Printed, Flexible and Hybrid MoS_2_-PVA Nanocomposite based Memristive Device Fabricated by Reverse Offset. *Sci. Rep.* **6**, 36195; doi: 10.1038/srep36195 (2016).

**Publisher’s note:** Springer Nature remains neutral with regard to jurisdictional claims in published maps and institutional affiliations.

## Supplementary Material

Supplementary Information

## Figures and Tables

**Table 1:** Comparison of MoS_2_-PVA nanocomposite based memristive device with already reported memory devices based on PVA nanocomposites.

Active Layer Materials	Voltage Sweep	Off/On ratio	Current Compliance	Endurance	Retention	Mechanical Robustness	Bend-ability	Ref.
MoS_2_-PVA	−4 to +4	1.28 × 10^2^	60 uA	1000	10^5^ s	1500 cycles	2 mm	
PVA-PbS	−30 to +30	<10	30 uA	—	—	—	—	35
AgNW-PVA	−10 to +10	~10	10 mA	160	—	—	—	36
PEDOT:PSS-PVA	−2 to +2	~10^2^	100 mA	—	36 × 10^4^ s	—	—	37
GNF–PVA	−7 to +2	~10^2^	1 mA	100	1 × 10^4^ s	—	—	38

It is evident from the illustrated results that MoS_2_ flakes play a vital role in improving the electrical properties of PVA based memory devices.

**Table 2:** Comparison of MoS_2_-PVA nanocomposite based memristive device with already reported memory devices based on nanocomposites of MoS_2_ semiconductor with other materials.

Active Layer Materials	Switching Voltage	Off/On Current Ratio	Retention Time	Endurance	Bending Diameter	Mechanical Robustness	Ref.
MoS_2_:PVA	3 V	1.28 × 10^2^	10^5^ s	1000 cycles	Up to 2 mm	1500 cycles	
MoS_2_-GO	<1.5 V	~10^2^	—	—	—	—	39
PVP-MoS_2_	~3.5 V	~10^2^	—	—	—	—	28
MoS_2_-P123	3–4 V	~5.5 × 10^2^	4 × 10^3^ s	—	—	—	40
ZIF-8/MoS_2_	~3.3 V	~7 × 10^4^	1.5 × 10^3^ s	—	—	—	41

It is evident from the illustrated results that PVA has enhanced the retention time and mechanical strength MoS_2_ based memory device.
