# Supplementary material for: Resistive Switching in All-Printed, Flexible and Hybrid MoS2-PVA Nanocomposite based Memristive Device Fabricated by Reverse Offset
Source: Sci Rep. 2016 Nov 4;6:36195. doi: 10.1038/srep36195 (PMC5095886; doi:10.1038/srep36195)
Supplement: Supplementary Information [file srep36195-s1.pdf]

Supplementary Information for

Resistive Switching in All-Printed, Flexible and Hybrid MoS_2-PVA Nanocomposite based Memristive Device Fabricated by Reverse Offset

Muhammad Muqeet Rehman[1], Ghayas Uddin Siddiqui[2], Jahan Zeb Gul[3], Soo-Wan KIM[4], Jong Hwan Lim[5], Kyung Hyun Choi[*]

[*, 1, 2, 3, 4, 5] Department of Mechatronics Engineering, Jeju National University, Jeju, Republic of Korea

*Correspondence to: amm@jejunu.ac.kr

[1]Email: muqeet@jejunu.ac.kr

[2]Email: ghayassiddiqui@jejunu.ac.kr

[3]Email: jahanzeb@jejunu.ac.kr

[4]Email: dolage89@jejunu.ac.kr

* Phone: +82-64-754-3713

* Fax: +82-64-752-3174

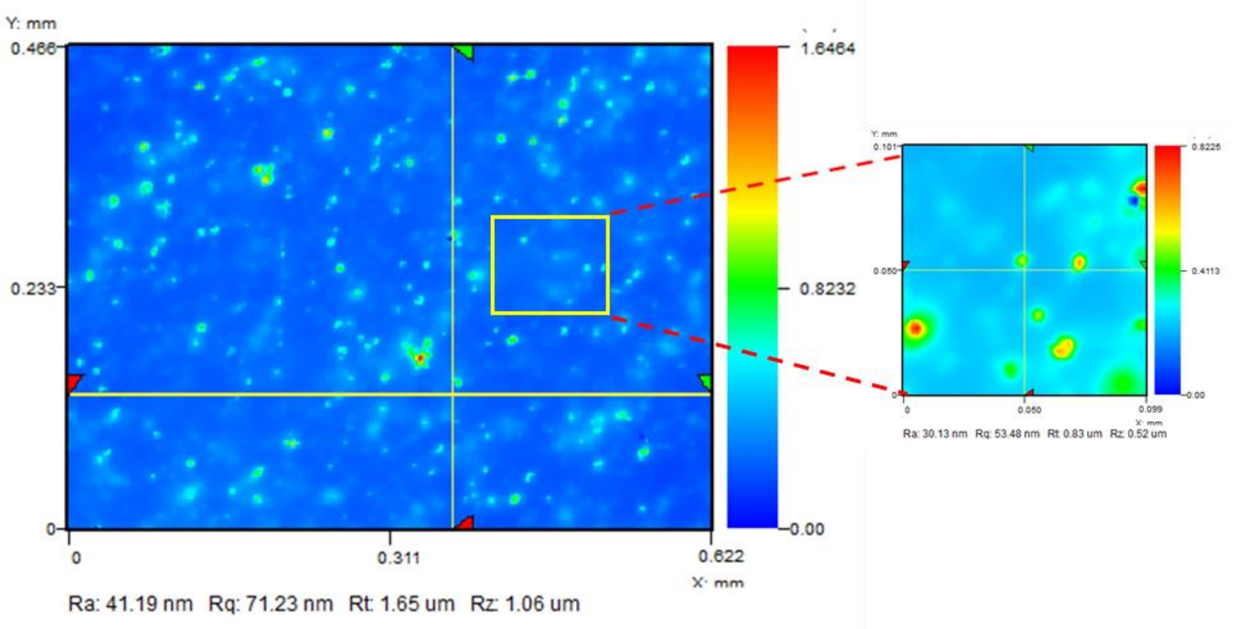

Figure S1 | 2D nanomap image displaying the surface morphology of as deposited active thin film of MoS_2-PVA nanocomposite by using EHD Atomization. The average roughness value is equal to 41.19 nm

2 D-Nano mapping was carried out by using NV-2000 (Universal) non-contact surface profiler with an accuracy of naoscale to measure the roughness in the thin film of MoS_2-PVA nanocomposite deposited by EHDA. This measurement was carried out in phase shifting interferometry mode. From the obtained 2D nanomap, it can easily be deduced that important parameters for measuring the roughness of thin film surface like arithmetic average height of peaks and valleys from the mean line (Ra), root mean square roughness (Rq), the maximum peak to valley height in the specific sampling length (Rt) and average peak to valley roughness (Rz) are 41.19 nm, 71.23 nm, 1.65 μm and 1.06 μm respectively Figure S1. In figure S the zoomed image of a specific region of the nanomap is also shown. The selected region shows even smaller surface roughness values with Ra, Rq, Rt and Rz equal to 30.13 nm, 53.48 nm, 0.83 μm and 0.52 μm respectively. These nanomap images shows that the resulted active thin film by EHDA possess good surface morphology.

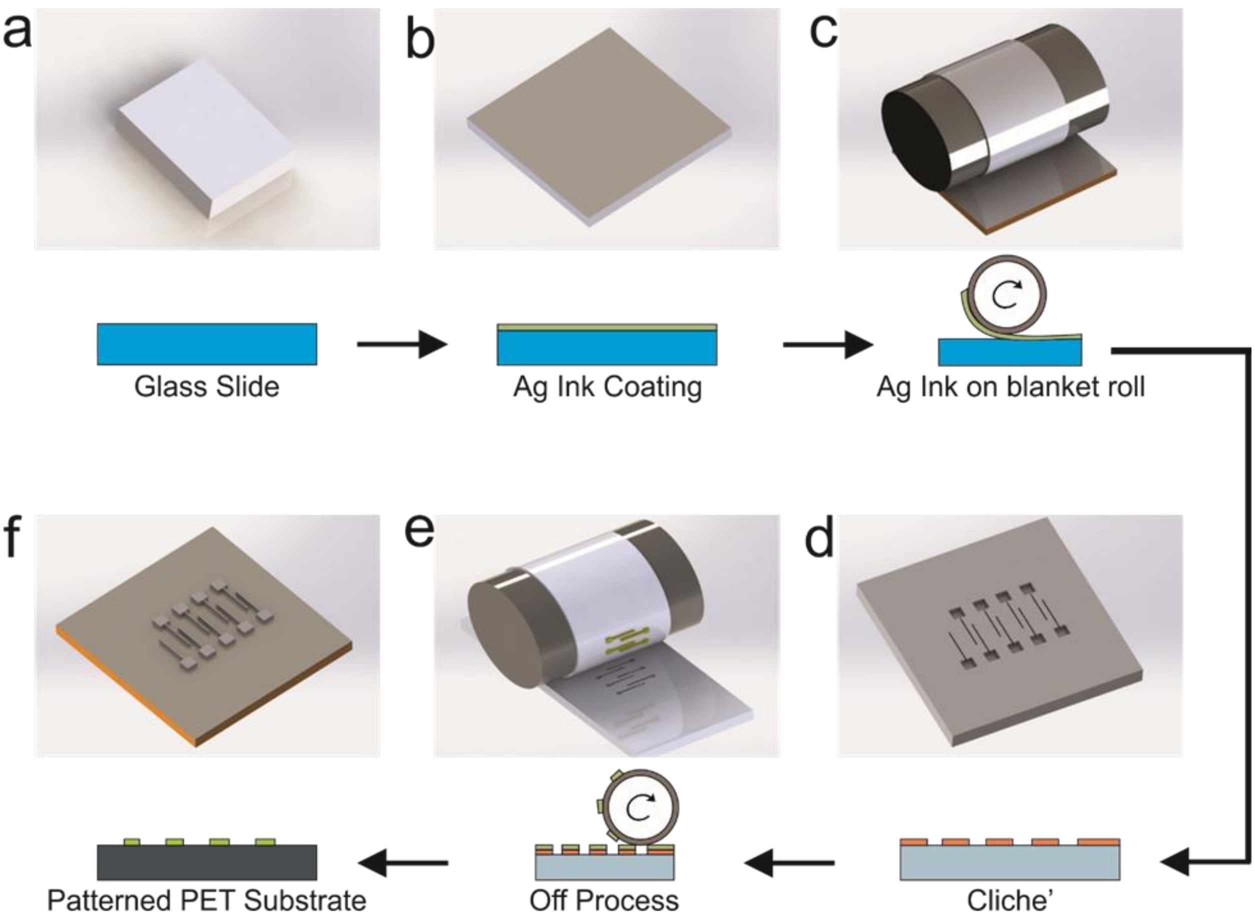

Figure S2 | 3D and 2D view of Reverse Off-Set schematic diagram illustrating the patterning of bottom Ag electrodes on flexible PET substrate a) Glass slide **b)** Ag nanoparticles ink spin coated on glass slide **c)** Ag ink being attached with the PDMS blanket by rolling the blanket roll over Ag coated glass slide **d)** Cliché showing the engraved patterns on its surface **e)** OFF Process taking place in which engraved patterns of cliché are being transferred onto PDMS blanket **f)** SET Process in which Ag electrodes are transferred from blanket roll onto the desired PET substrate

Accessories of reverse offset printing system include a glass slide to deposit ink, a roller enfolded with a blanket of highly hydrophobic poly-dimethylsilxane (PDMS) material, a cliché designed with the negative of electrode patterns engraved on its surface and finally the desired substrate on which the device has to be fabricated. In order to achieve high resolution micro patterns, large pressure and low speed are the desired parameters. To achieve quick prototyping for mass production without causing any damage, both parameters have to be optimized before performing experiments**.** Electrodes are printed at once in a single go.

Initially Ag ink was spin coated on a coating substrate (glass slide in our case) at 3000 rpm as shown in Fig. S2a-b. The blanket roll was coated with Ag ink by rolling it over the spin coated glass slide. A thin, yet uniform and continuous film of Ag nanoparticles ink was transferred on the PDMS blanket roll due to its high absorption coefficient as displayed in Fig S2c. A cliché design with the negative of desired patterns acutely carved on its surface in the form of trenches as shown in Fig S2d. Ink coated roll was moved in forward direction and pressed over the cliché with optimized speed and pressure. The patterns were transferred on blanket roll as all the unnecessary Ag ink was left on the cliché surface due to its superior adhesion coefficient as compared to the blanket roll (Off Process) as illustrated in Fig S2e. In the final step, the roll was moved in forward direction where it was pressed over the desired flexible PET substrate and Ag electrode patterns were shifted on it (Set Process) as displayed in Fig S2f. The area of the blanket roll known as nip which remains in continuous contact with the plate, cliché or desired substrate is extremely important as transfer of ink from or to the blanket roll occurs in this area[1]. Adhesion and cohesion of each surface is also an important parameter which was optimized by controlling the pressure and speed of the blanket roll. Ink adheres to the roll and separated from the surface at the end of contact. Extremely fine Ag electrodes with 100 um width and excellent resistivity of 0.2 ohm-cm were achieved after sintering at 110 °C for 1 hour in a furnace. Optical images of as fabricated bottom electrodes at different resolutions are shown in Figure | S3. Optical image of reverse offset printing system and bottom electrodes on fabricated device is shown in Figure | S4.

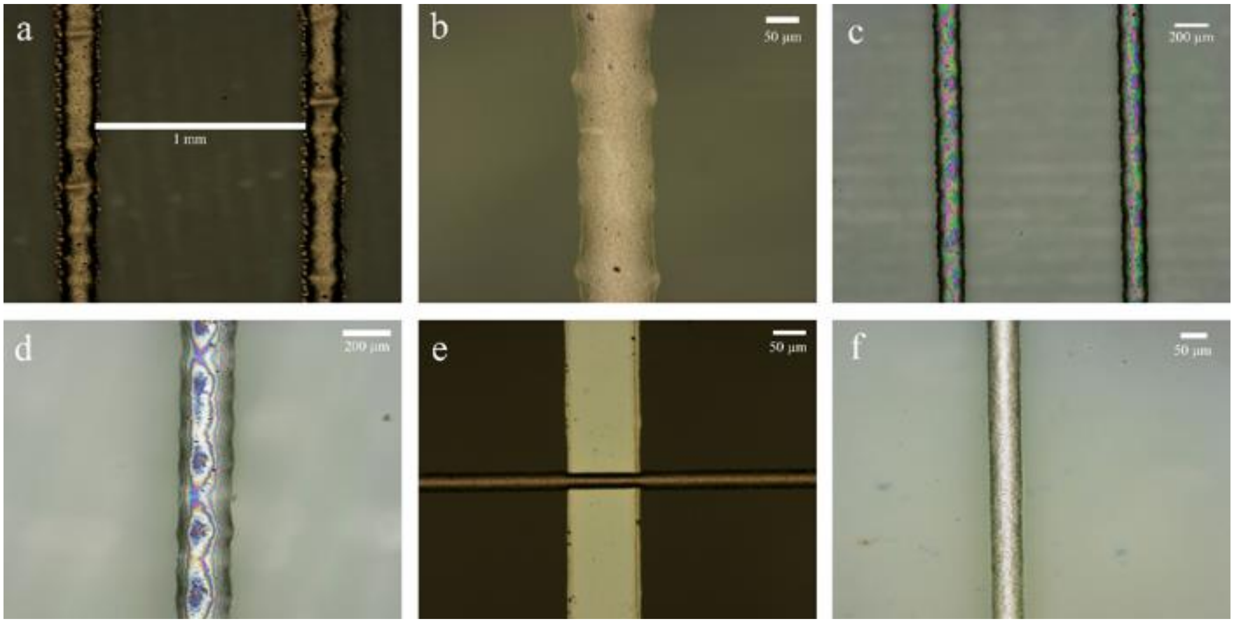

Figure S3 | Optical images of bottom and top Ag electrodes displaying their high quality patterned by using reverse offset and EHD patterning systems respectively a) gap between side by side patterned bottom electrodes **b)** Magnified image of a single bottom electrode **c)** Two consecutive bottom electrodes with a thin film of MoS_2-PVA nanocomposite coated on them by EHDA **d)** Magnified image of a single bottom electrode with a thin film of MoS_2-PVA nanocomposite coated on it by EHDA **e)** Image of top electrode bisecting the bottom Ag electrode **f)** Magnified image of top electrode displaying its remarkably uniform surface

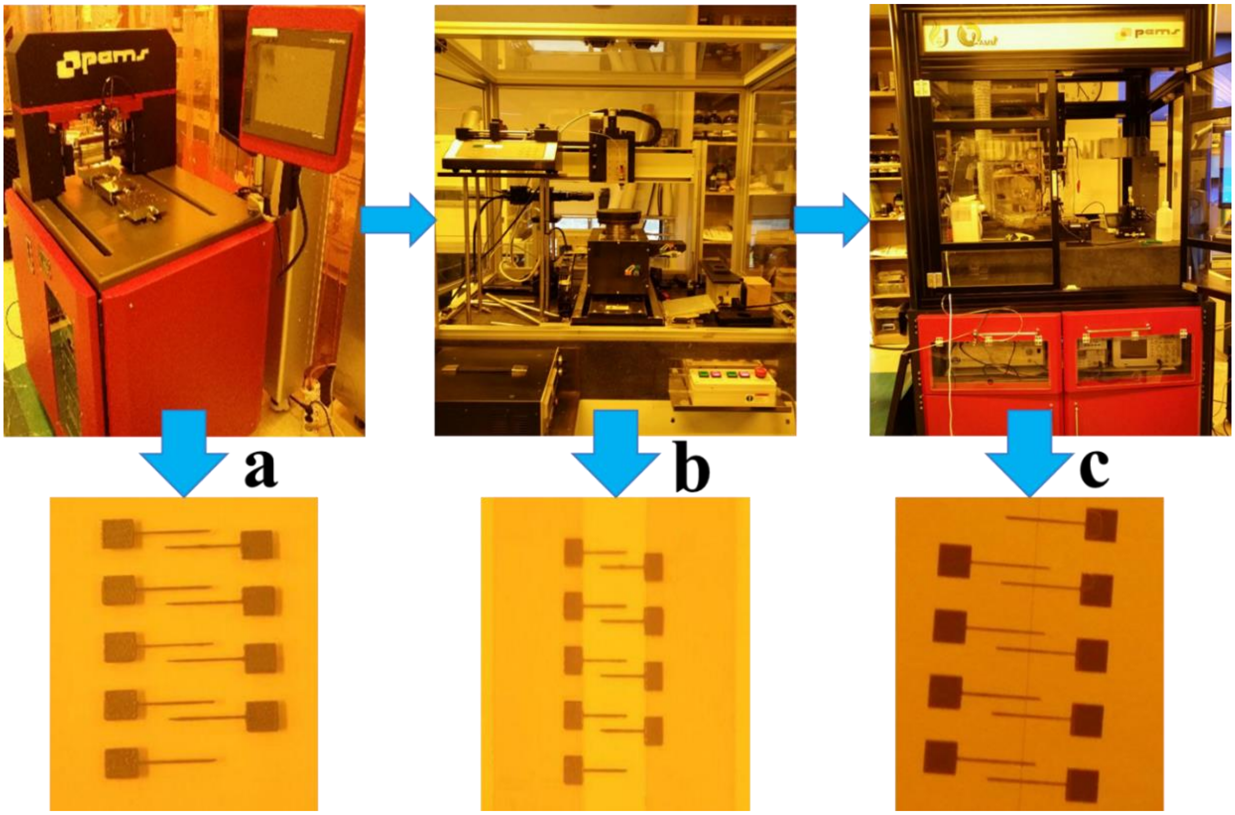

Figure S4 | Optical images of state of the art printing systems used for the fabrication of flexible Ag/MoS_2-PVA/Ag memory device a) Reverse offset patterning system **b)** EHD Atomization for spray coating of MoS_2-PVA nanocomposite **c)** EHD patterning system

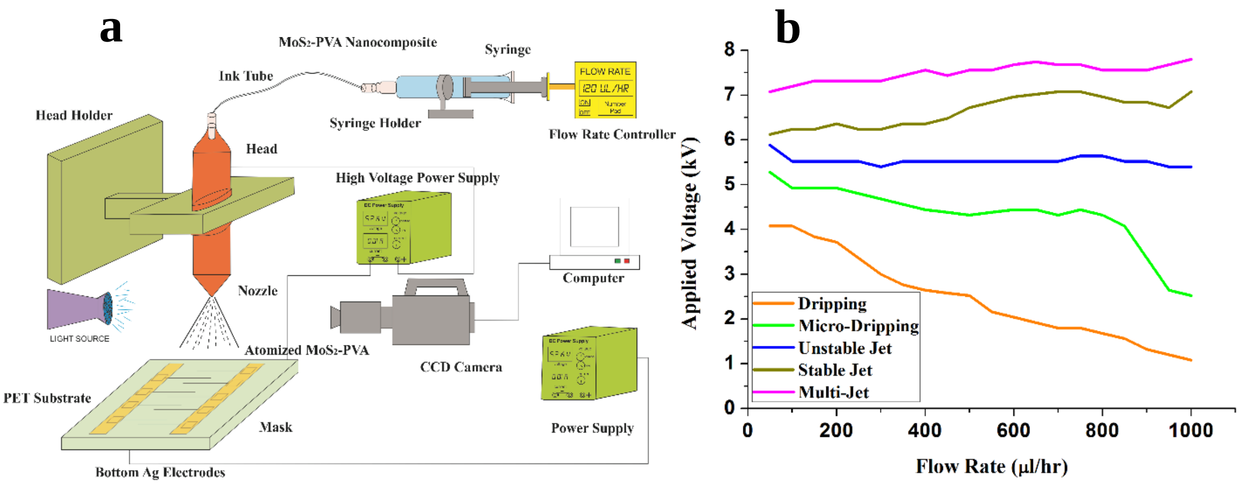

Figure S5: a) Schematic Diagram of EHD atomization process with intensive labelling of each part **b)** Operating Envelope of EHD Atomization for the deposition of MoS_2-PVA nanocomposite

Parameter	Optimized Value
Flow Rate	100 ul/h
Applied Voltage	2.9 kV
Nozzle to substrate distance	10 mm
Stage Speed	6 mm/s
Nozzle Diameter	200 um
Total No of Spraying Passes	6

Table S1 | Optimized values of various parameters to control the film thickness and uniformity during EHD Atomization of MoS_2-PVA nanocomposite

Mode of Deposition	Voltage Range
Dripping	0- 4.9 kV
Micro Dripping	4.9-5.5 kV
Unstable Cone-Jet	5.5-6.2 kV
Stable Cone-Jet	6.2-7.1 kV
Multi-stable Cone-Jet	> 7.1 kV

Table S2 | Various Modes of Deposition along with the Range of Applied Voltage Difference from High- Voltage Power Supply during EHD Atomization of MoS_2-PVA nanocomposite

Parameter	Optimized Value
Nozzle to Substrate Distance	5 um
External Nozzle Diameter	30 um
Nozzle Speed	0.3 mm/s
Applied Pressure	64.6 kPa

Table S3 | Optimized condition and their set values during the deposition of top Ag electrode through EHD Patterning Process of Top Ag Electrode

Reference:

1. Kang, D. *et al.* Investigation on synchronization of the offset printing process for fine patterning and precision overlay. *J. Appl. Phys.* **115,** (2014).
